# A genetically and functionally diverse group of non-diazotrophic *Bradyrhizobium* spp. colonizes the root endophytic compartment of *Arabidopsis thaliana*

**DOI:** 10.1186/s12870-018-1272-y

**Published:** 2018-04-11

**Authors:** Martinus Schneijderberg, Lucas Schmitz, Xu Cheng, Sharon Polman, Carolien Franken, Rene Geurts, Ton Bisseling

**Affiliations:** Department of Plant Sciences, Laboratory of Molecular Biology, Wageningen University, Droevendaalsesteeg 1, 6708 PB Wageningen, The Netherlands

**Keywords:** *Bradyrhizobium*, Arabidopsis, Root colonization, Endophytic compartment

## Abstract

**Background:**

Diazotrophic *Bradyrhizobium* spp. are well known for their ability to trigger nodule formation on a variety of legume species. In nodules, *Bradyrhizobium* utilizes plant-derived carbohydrates in exchange for fixed nitrogen. The genes essential for the nodulation and nitrogen-fixation trait are clustered in a genomic region, which is known as the ‘symbiotic island’. Recently, novel non-diazotrophic *Bradyrhizobium* spp. have been found to be highly abundant in soils, suggesting that these species can also have a ‘free-living’ life history. However, whether non-diazotrophic *Bradyrhizobium* spp. can live in association with plants remains elusive.

**Results:**

In this study, we show that *Bradyrhizobium* spp. are common root endophytes of non-legume plant species – including *Arabidopsis thaliana* (Arabidopsis) – grown in an ecological setting. From a single Arabidopsis root, four *Bradyrhizobium* sp. strains (designated MOS001 to MOS004) were isolated. Comparative genome analysis revealed that these strains were genetically and functionally highly diverse, but did not harbour the nodulation and the nitrogen fixation gene clusters. Comparative colonization experiments, with MOS strains and nitrogen-fixing symbiotic strains, revealed that all tested *Bradyrhizobium* spp. can colonize the root endophytic compartment of Arabidopsis.

**Conclusion:**

This study provides evidence that both diazotrophic and non-diazotrophic *Bradyrhizobium* spp. colonize the root endophytic compartment of a wide variety of plant species, including the model species Arabidopsis. This demonstrates that plant roots form a major ecological niche for *Bradyrhizobium* spp., which might be ancestral to the evolution of the nodulation and nitrogen-fixation trait in this genus.

**Electronic supplementary material:**

The online version of this article (10.1186/s12870-018-1272-y) contains supplementary material, which is available to authorized users.

## Background

Plants can develop relationships with soil bacteria, which vary from loose associations in the rhizosphere up to intimate inter- and intracellular hosting in plant tissues. An eminent and well-studied example of an intimate interaction is the relationship between legumes and a paraphyletic group of nitrogen-fixing α- and β-proteobacteria, collectively known as rhizobia [1]. Legumes can form special organs on their roots – known as nodules – that facilitate rhizobia to convert atmospheric nitrogen into ammonium, which subsequently can be utilized by the plant [2]. The bacterial genes underlying this nitrogen-fixing nodule symbiosis are the nodulation (*nod* and *nol*) and nitrogen fixation (*nif* and *fix*) genes. These genes are organized in one or more clusters on the genome or symbiotic plasmid(s). The *nod*/*nol* and *nif*/*fix* genes – as identified in the diverse range of rhizobial genera – are highly homologous and it is therefore widely accepted that these symbiotic genes were transmitted by horizontal gene transfer [3]. This suggests that the lifestyle of ancestral rhizobium species was unrelated to nodule symbiosis.

One of the most profound clades of symbiotic rhizobia is the genus *Bradyrhizobium* (within the family of the Bradyrhizobiaceae). Defined as “slow-growing, non-acid-producing root nodule bacteria of leguminous plants” [4], this genus encompasses now 37 species [5]. The type strains of these species all nodulate and have a wide range of legumes (including *Glycine max*; soybean), and the non-legume *Parasponia* spp. as host [6, 7]. The genomes of *Bradyrhizobium* spp. are relatively large (7–10 Mb), and the nodulation (*nod/nol*) and nitrogen fixation (*nif/fix*) gene clusters are located on a symbiotic island within the genome [8].

Recent studies revealed that *Bradyrhizobium* spp. are also highly abundant in soils in absence of legume plants. At first, evidence came from culture-independent studies on the bacterial communities in soils and rhizospheres of oak trees. These communities harboured large populations of *Bradyrhizobium* spp. [9–11]. Additional evidence for the abundance of *Bradyrhizobium* spp. in absence of legumes was found in coniferous forest soils of North America [12]. Through quantitative population genomics and whole-genome sequencing, it was shown that the identified *Bradyrhizobium* spp. lack the *nod/nol* and *nif/fix* gene clusters, and consequently are incapable to nodulate legumes or to fix atmospheric nitrogen. Instead, these strains possess multiple gene clusters affiliated with complex carbon metabolism and aromatic compound degradation. Likewise, two *Bradyrhizobium* spp. strains isolated from soils of a bare fallow field and a grassland did not possess the symbiotic island [13]. Together, these studies indicate that *Bradyrhizobium* spp. can not only live in symbiosis with legumes, but can also have a ‘free-living’ life history. However, it remains unknown whether such non-nitrogen fixing *Bradyrhizobium spp.* can have intimate relationships with plants.

As nodulating and nitrogen-fixing *Bradyrhizobium* spp. can establish an intracellular lifestyle with their legume hosts, we hypothesised that *Bradyrhizobium* spp. lacking the symbiotic island can live in a close intimate association with plants. In this study, we investigated whether such *Bradyrhizobium* spp. occur on or inside the roots of non-legumes in a natural ecosystem. We made use of a dataset from an ecological test field in The Netherlands at the Veluwe area called the Mossel, where we previously studied the root microbiome of nine species, among which *Arabidopsis thaliana* (Arabidopsis) (Schneijderberg et al., in preparation).

## Results

### *Bradyrhizobium* spp. colonize plant roots

**Fig. 1:**
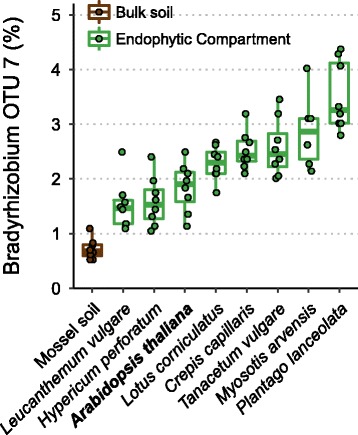
16S rDNA amplicon sequencing reveals high abundance of *Bradyrhizobium* in all tested plant species. The plant species (listed in Table 1) were grown for 7 weeks on an experimental plot in the Veluwe Mossel area (The Netherlands). Shown is the relative abundance of *Bradyrhizobium* OTU 7 in the root endophytic compartment. Each dot represents one replicate (*n* = 8)

To determine whether *Bradyrhizobium* species can colonize non-legume plant roots, we analysed a 16S rDNA amplicon dataset from a separate but simultaneous experiment (Schneijderberg et al. in preparation). This dataset comprises a field experiment at the Veluwe area (a region called the Mossel) in The Netherlands and includes nine plant species representing six taxonomic orders (Table 1). Eight of these species are non-legumes, among which is Arabidopsis (accession Mossel; Msl). The bacterial communities of the root endophytic compartments of these species have been determined by 16S rDNA amplicon sequencing, after growing for 8 weeks in the summer of 2016 on an experimental plot in the Mossel (Schneijderberg et al. in preparation). Analysis revealed four operational taxonomic units (OTUs) affiliated with the genus *Bradyrhizobium*, of which one OTU (#7) was highly abundant in both the soil (0.5%) and the plant root endophytic compartment (Fig. 1). This OTU was not only abundant in the root endophytic compartment of the legume *Lotus corniculatus*, but also in root samples of all the eight non-legumes, ranging from 1.5% in *Leucanthemum vulgare* to more than 3% in *Plantago lanceolata* (Fig. 1). The widespread abundance of *Bradyrhizobium* spp. in the root endophytic compartment of all studied plant species indicates that root colonization by strains of this genus is a generic phenomenon in the Mossel area.

**Table 1:** Plant species native to the Veluwe Mossel area in the Netherlands that were used in the 16S rDNA meta amplicon analysis

Species	Order	Common name
*Arabidopsis thaliana*	Brassicales	Thale cress / Arabidopsis
*Crepis capillaris*	Asterales	Smooth hawksbeard
*Hypericum perforatum*	Malpighiales	St John’s wort
*Leucathemum vulgare*	Asterales	Ox-eye daisy
*Lotus corniculatus*	Fabales	Bird’s-foot trefoil
*Myosotis arvensis*	Boraginales	Field forget-me-not
*Plantago lanceolata*	Lamiales	Narrowleaf plantain
*Tanacetum vulgare*	Asterales	Tansy

To determine whether *Bradyrhizobium* spp. are root endophytes in other ecosystems as well, we analysed publicly available 16S rDNA amplicon datasets. This showed that *Bradyrhizobium* OTUs are present in the endophytic compartment of a variety of plants, including Arabidopsis, rice (*Oryza sativa*) and maize (*Zea mays*) (Table 2). This strongly supports that *Bradyrhizobium* spp. are common endophytes in plant roots. However, whether the strains that make up the *Bradyrhizobium* OTUs in those studies possess the symbiotic island, remains elusive.

**Table 2:** *Bradyrhizobium* identified in root endophytic compartment

Species	Relative Abundance (%)	Reference
*Arabidopsis thaliana*	1	[44]
*Arabidopsis thaliana*	3	[45]
*Oryza sativa*	1	[46]
*Zea mays*	0,3	[47]

### Arabidopsis endophytic *Bradyrhizobium sp.* MOS strains lack the nitrogen fixation trait

An OTU based on the 16S rDNA V4 region can represent multiple strains or even species. To determine whether there are multiple strains belonging to OTU 7 and whether these strains possess the nodulation and nitrogen fixation gene clusters, we set out on an isolation and characterization strategy for *Bradyrhizobium* spp. From a single Arabidopsis root grown in the Mossel experimental plot, we isolated 102 strains that showed growth characteristics of *Bradyrhizobium*. These strains were classified in 12 groups based on DNA-fingerprinting using BOX-PCR (Additional file 1). Next, we sequenced the 16S rDNA locus of a representative strain of each group. This revealed four strains (representing 85% of the isolates) that matched with *Bradyrhizobium* in the RDP database [14] (Additional file 1). The V4 region of the 16S rDNA gene of these four strains showed more than 99% identity to the consensus sequence of OTU 7, indicating that these isolates represent this OTU. We named these strains *Bradyrhizobium* sp. MOS001 to *Bradyrhizobium* sp. MOS004, after the area from which they originated. A fifth strain designated MOS005, classified as *Bradyrhizobium* based on the 16S rDNA gene, was also sequenced. However, since the full genome sequence could not ensure that this was a *Bradyrhizobium* sp., we chose to exclude it from the analysis (Additional file 1).

To determine whether these *Bradyrhizobium* spp. MOS strains possess the nitrogen fixation trait, we assembled draft genome sequences based on a 150 base pair paired-end library sequenced on the Illumina HiSeq platform. This resulted in draft assemblies ranging in size from 7.6 (MOS003) to 9.1 Mb (MOS001), representing 6879 (MOS003) to 8622 (MOS001) annotated gene models (Additional file 2). Such genome sizes, as well as the corresponding GC contents (64–66%), are comparable to previously sequenced *Bradyrhizobium* spp. genomes [12, 13, 15, 16]. To verify the completeness of the draft genome assemblies of *Bradyrhizobium* sp. MOS strains, Busco analysis was conducted [17]. This revealed full-length presence of 97.6% in MOS004 and 99.1% in the other MOS strains of the Rhizobiales test set of 686 genes, indicating close to complete genome coverages (Additional file 2).

**Fig. 2:**
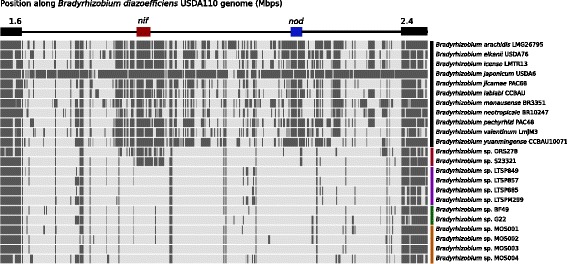
Reciprocal best blast hit analysis shows that *Bradyrhizobium* sp. MOS strains lack the symbiotic island. On top a schematic representation of the symbiotic reference strain *B. diazoefficiens* USDA110, with in black the conserved regions, in red the *nif/fix* gene cluster and in blue the *nod/nol* gene cluster. On the right, the strain name (color-coded as followed. Black: the type strains from Fig. 3; red: strains possessing only the *nif/fix* gene cluster; purple: the non-symbiotic LTSP strains [12]; green: the non-symbiotic strains G22 and BF49 [13]: orange, the MOS strains. Dark grey bar means a hit on the reference genome, with a coverage of ≥50% and a similarity of ≥70%. The *Bradyrhizobium* sp. MOS strains do not have a hit in the *nod/nol* or *nif/fix* gene clusters, suggesting absence of the symbiotic island. As expected, the LTSP strains as well as the G22 / BF49 strains show no hits in the symbiotic genes either

To determine whether *Bradyrhizobium* sp. MOS strains possess nodulation and/or nitrogen fixation genes we used a reciprocal best blast hit algorithm with the nitrogen-fixing symbiont *Bradyrhizobium diazoefficiens* strain USDA110 as reference [16]. This revealed that the four *Bradyrhizobium* sp. MOS strains do not have any hit in either the *nod*/*nol* gene cluster or the *nif/fix* gene cluster (Fig. 2). Therefore we conclude that *Bradyrhizobium* sp. MOS strains are incapable of nodulation and nitrogen fixation.

### Arabidopsis endophytic *Bradyrhizobium* sp. strains are highly diverse

**Fig. 3:**
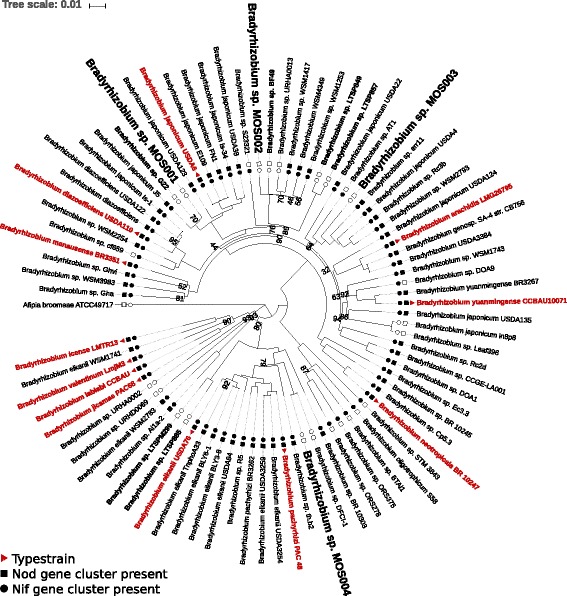
Phylogenetic analysis shows that the *Bradyrhizobium* sp. MOS strains are genetically diverse. The tree was constructed using a concatenated nucleotide alignment of 31 AMPHORA genes [18]. For each strain, the presence of the *nod/nol* gene cluster (filled squares) and the *nif/fix* gene cluster (filled circles) are indicated. A red triangle means that the particular strain is a type strain for that species [5]. The *Bradyrhizobium* sp. MOS strains fall into four different clades. *Afipia broomae* ATCC49717 was used as outgroup

Based on the genetic fingerprinting the four isolates are different. In order to get insight in the taxonomic diversity represented by the four isolated *Bradyrhizobium* sp. MOS strains, the phylogenetic relation was determined. Since we obtained full genome sequences, we based our phylogenetic analysis on the nucleotide sequences of 31 AMPHORA genes [18], which comprise a set of highly conserved marker genes. We aligned these genes against all *Bradyrhizobium* spp. for which genomes have been published, including non-symbiotic strains identified in forest areas (designated LTSP [12]) and grassland (designated G22 and BF49 [13]). Subsequently, the phylogenetic relation was reconstructed (Fig. 3). The topology of the phylogenetic tree is in line with previously reported phylogenies based on single or multiple sequence alignments [12, 19, 20]. The *Bradyrhizobium* sp. MOS strains represent four distinct lineages within the genus (Fig. 3). *Bradyrhizobium* sp. MOS001 and MOS002 belong to different lineages of a clade represented by the type strain *B. japonicum* USDA6 and cluster with the two non-symbiotic strains isolated from grassland (namely *Bradyrhizobium* sp. G22 and BF49, respectively). *Bradyrhizobium* sp. MOS003 falls within a large clade, which includes the type strains *B. arachidis* LMG26795 and *B. yuanmingense* CCBAU10071, whereas *Bradyrhizobium* sp. MOS004 shows high sequence homology to *Bradyrhizobium* sp. DFCI-1, within the *B. elkanii* USDA76 clade.

**Fig. 4:**
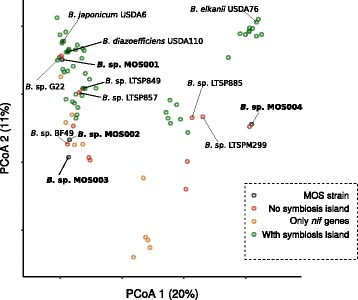
PCoA on dissimilarities matrix shows that the *Bradyrhizobium* sp. MOS strains are functionally diverse. Plotted are the first two principal coordinates, which explain 20% and 11%, respectively. Each circle in the plot represents one functional profile. Green: strains containing the symbiotic island; orange: strains only possessing the nitrogen fixation gene cluster; red: strains lacking the symbiotic island; black: *Bradyrhizobium* sp. MOS strains, also lacking this island

Since the four strains originate from a single Arabidopsis root, we questioned whether the isolates are functionally similar, despite their genetic divergence. We used a previously published custom R pipeline [21] to predict the functional groups of all the open reading frames in each of the isolates using the KEGG Orthology (KO) database [22]. After determining presence and absence of the KO groups and creating a pair-wise dissimilarity matrix using a binary distance measure, we plotted each functional profile along the first two Principal Coordinates. This resulted in a PCoA in which the distances between the species resemble the phylogenetic relationship of the strains (Fig. 4). Along the first two principal coordinates, the *Bradyrhizobium* sp. MOS001, MOS002 and MOS003 strains cluster relatively close to *B. diazoefficiens* USDA110 and *B. japonicum* USDA6, while MOS004 are relatively close to the *B. elkanii* clade. This indicates that the group of MOS isolates is not only genetically, but also functionally diverse.

### The MOS isolates recolonize the Arabidopsis root

**Fig. 5:**
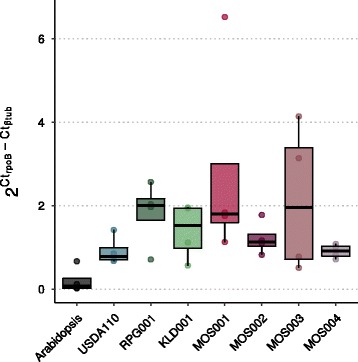
qPCR-based quantification shows *Bradyrhizobium* spp. colonization of Arabidopsis roots. On the y-axis an approximation of the number of bacterial genome copies per plant genome copy. This is calculated by exponentiating 2 by the difference between the Ct values of rpoB (targeting bacterial DNA) and Btub (targeting B tubulin in Arabidopsis). Two plant roots grown in autoclaved river sand supplemented with bacteria were pooled, a total of four replicates per treatment was measured. Arabidopsis: mock treated plants. USDA110: *Bradyrhizobium diazoefficiens* USDA110. RPG001: *Bradyrhizobium* sp. isolated from *Chamaecrista mimosoides* nodule. KLD001: *Bradyrhizobium* sp. isolated from *Parasponia andersonii* nodule. These strains possess the symbiotic island

We aimed to determine whether *Bradyrhizobium* sp. MOS001, MOS002, MOS003 and/or MOS004 have the capacity to colonize a plant root endophytic compartment in an experimental system. Furthermore, we questioned to what extent this colonization capacity is similar to the colonization capacity of diazotrophic *Bradyrhizobium* spp. To investigate this, we set up a growth assay in where 7 days-old in vitro grown Arabidopsis seedlings (Accession Msl) are transplanted into autoclaved river sand inoculated with a *Bradyrhizobium* strain to a density comparable with the density of *Bradyrhizobium* in the native Mossel soil, which is approximately 2.5 × 10^5^ cells per gram of soil. After 2 weeks, plants were analysed. None of the four *Bradyrhizobium* sp. MOS strains, nor any of 3 tested nitrogen-fixing symbiotic strains affected the fresh weight of the Arabidopsis plants (Additional file 3). To quantify root colonization of the different strains, we used qPCR on the highly conserved bacterial *rpoB* gene and normalized that against tubulin binding co-factor C of Arabidopsis [23]. While detection of bacterial DNA in the roots of non-inoculated control plants was close to zero, DNA of all strains was detected in the root endophytic compartment samples in the quantity of approximately 2 bacterial genome copies for every plant genome copies (Fig. 5). Comparing the four *Bradyrhizobium* sp. MOS strains with the three nitrogen-fixing symbionts did not reveal a significant difference in colonization capacity. This shows that symbiotic and non-symbiotic *Bradyrhizobium* strains colonize the Arabidopsis root equally well in our growth system (Fig. 5).

## Discussion

Here we showed that the endophytic compartment of a variety of non-legume plants – including Arabidopsis – is colonized by a diverse range of non-nitrogen fixing *Bradyrhizobium* species. Earlier studies revealed that *Bradyrhizobium* species are enriched in rhizosphere soils of non-legumes. For example, in a recent study *Bradyrhizobium*, but also *Rhizobium* and *Bulkholderia* OTUs were found in the core rhizosphere microbiome of a tropical chronosequence comprising 31 plant species [24]. This is in line with a growing body of data that suggests rhizobium-plant interactions are evolutionary conserved and that a non-endosymbiotic interaction may be widespread [12, 13, 24]. Whether the rhizobia identified in those studies possess the symbiotic genes remains to be tested. The fact that the *Bradyrhizobium* sp. MOS strains that we identified in the root endophytic compartment of Arabidopsis, and other non-legume species, do not have the symbiotic island, shows that the interaction is independent of the known nodulation and fixation genes. Also, we conclude that the colonization of the endophytic compartment by *Bradyrhizobium* spp. is a common trait and that these *Bradyrhizobium* spp. most probably have a wide host range.

The four *Bradyrhizobium* sp. MOS strains were isolated from a single Arabidopsis root. Surprisingly, we found that they are genetically highly diverse. Using 31 conserved amphora genes, we analysed the phylogeny of the MOS strains and all publicly available Bradyrhizobium isolates. Based on analysis by the Average Nucleotide Identity software [25], we conclude that MOS001 represents most probably a *B. japonicum* strain as it shows ~ 95% sequence identity with the type strain USDA6. In contrast, MOS002, MOS003 and MOS004 the sequence similarity with known type strains is less than 95%, and therefore these strains possibly represent new species. The fact that the isolates have highly diverse functional profiles suggests that the absence of the symbiotic island is not linked to a specific non-symbiotic lifestyle, but that the absence of the symbiotic island is widespread and that these non-diazotrophic species have diverse life strategies.

Re-inoculation experiments on Arabidopsis did not show any plant growth effects of any of our strains under the conditions that we tested. However, it is well possible that within the setting of a complex microbiome, plants as well as the microbes benefit from the close association. We showed that the isolated *Bradyrhizobium* sp. MOS strains colonize roots with an efficiency comparable to nitrogen-fixing symbionts. In line with this, we argue that an endophytic lifestyle is likely a common trait of *Bradyrhizobium* sp. Previously, it has been shown that the nodulation and nitrogen fixation genes have been distributed by horizontal gene transfer within the various genera that can establish a nodule symbiosis with legumes [3, 26]. Therefore it seems probable that *Bradyrhizobium* strains with an endophytic life style acquired the *nod/nol* and *nif/fix* genes by horizontal gene transfer and in this way obtained the specialized nitrogen-fixing symbiotic lifestyle.

## Conclusions

This study shows that *Bradyrhizobium* spp. colonize the root endophytic compartment of a wide variety of plant species, including the model species Arabidopsis. Four isolates from a single Arabidopsis root have high genetic and functional variation, but all lack the genes for nodule formation and nitrogen fixation. These non-diazotrophic *Bradyrhizobium* sp. MOS strains, as well as diazotrophic strains, re-colonize the root endophytic compartment of Arabidopsis. Taken together, this study demonstrates that plant roots form a major ecological niche for *Bradyrhizobium* spp., which might be ancestral to the evolution of the nodulation and nitrogen-fixation trait in this genus.

## Methods

### Soil collection and field experiment

Soil was collected in May 2016 at the Mossel area at the ‘Hoge Veluwe’ in the Netherlands (coordinates: N52°03′35.5″ E5°45′06.4″), from four different spots within a radius of 100 m. If there was any vegetation present, the top 5–10 cm soil was removed. The soil was homogenized and all detritus was removed. The soil was kept in a cold room at 4 °C until use. All plant species were sterilized in 4× diluted household bleach for 10 min, washed seven times with sterile MQ water and transferred to plates with a wet filter paper, placed at 4 °C for 48 h and then moved to a 21 °C incubator in the dark. Seeds of Arabidopsis Msl also got a short rinse with 70% ethanol and the seeds of *L. corniculatus* and were treated 2 min with H_2_SO_4_ before exposure to the household bleach solution. Seeds of *T. officinale*, *T. vulgare*, *L. vulgare* and *M. arvensis* were placed directly in the 21 °C incubator without cold treatment. Mossel soil was placed in a tray with 3 × 3 cm pots and watered. To remove the endogenous seed population, the tray was placed in the greenhouse for 2 days. After weeding, the sterile seedlings on the plates were transplanted to the tray with Mossel soil and after 7 days the plants including the soil were planted into to the Mossel field.

### Plant harvesting and DNA isolation of the microbial community

After 6 weeks of growth in the field, plants were excavated using a small shovel 3–4 cm around the base of the plant. Consequently, the holes were 6–8 cm wide and around 10 cm deep. After transporting the plants (including the clod) to the laboratory, we applied the harvesting protocol as described before [27]. Four individual plants were pooled into one sample. DNA was isolated from soil and endophytic compartment samples using the Mo Bio PowerSoil kit (Qiagen) and the Fast DNA Spin Kit for Soil (MP Biomedicals) respectively. Quality and quantity of the DNA was checked by nanodrop and gel electrophoresis. Around 400 ng was sent for 16S rDNA sequencing at Beijing Genomics Institute (BGI).

### 16S rDNA amplicon sequencing and data processing

Using primers 515F and 806R [28], the V4 region was sequenced at BGI on the HiSeq2500 sequencing platform (Illumina). Raw data from BGI was processed using a previously reported custom implementation [29] of QIIME [30] with minor modifications which are described in detail in Schneijderberg et al. (in preparation). In short, reads were quality filtered and filtered for chimeras using ChimeraSlayer. Using a 97% identity threshold, de novo OTUs were determined, which were taxonomically assigned using the RDP classifier with the GreenGenes database. OTUs related to mitochondrial and chloroplast sequences were removed, as were the OTUs that did not have 25 reads in at least 5 samples. To obtain relative abundance of *Bradyrhizobium*, reads from OTUs matching *Bradyrhizobium* were added up per sample, and then divided by the total number of reads of that sample after filtering rare taxa.

### Arabidopsis growth assay

Sterile Arabidopsis Msl Seeds were transferred to ½ MS-plates and incubated at 21 °C with a 16 h/8 h photoperiod. After 7 days, roots were ca. 3 cm in length, and transplanted to pots which were prepared as follows: bacterial cultures were spun down, washed with 10 mM MgSO_4_ and added to 15 ml of ¼ Hoagland’s medium [31]. This medium was added to 4 × 4 cm pots filled with sterile river sand. The soil humidity was set at 70% of the water holding capacity with a bacterial cell density of 4 × 10^5^ cells per gram of sterile sand. The axenic control was treated with ¼ Hoagland’s only. During the grow period, 5 individual pots were weighed to estimate water loss and if necessary plants were irrigated with sterile water. After 2 weeks, the plants were carefully dug out from the sand and the shoot was separated from the root. Shoot biomass was immediately measured. The roots underwent the harvesting protocol previously described [27] and were stored at − 80 °C until DNA was extracted as follows: roots were grinded with metal beads in a Tissue Lyzer after which 500 μL of CTAB buffer was added. Samples were incubated for 30 min at 65 °C. Next, 500 μL of chloroform was added and the sample was spun down for 5 min at 14,000 g. Then, the upper phase was transferred to a new Eppendorf tube containing 400 μL of isopropanol. The sample was incubated at − 80 °C for 1 h. After incubation, the mixture was spun down for 10 min at 14,000 g and the DNA pellet was washed with 500 μL of 70% ethanol. After another step of centrifugation, the ethanol was removed and the DNA pellet was dried for 15 min at room temperature. Finally, the pellet was resuspended in 50 μL of MilliQ water and stored at − 2 °C until further use.

### Quantitative PCR

Primers targeting the *rpoB* gene in *Bradyrhizobium* spp. (rpoB_Fw1; 5’-CGCTGAAGAACCTCGACGAAGCC-3′ and rpoB_Rv1; 5’-CGGCGTGATCTTGCCGACCAG-3′) were designed using Geneious 8.1. The Arabidopsis B-tubulin gene primers (Forward: 5’-AGAAAACCGGAAACGAGAGC-3′ and Reverse 5’-ACAAGACACTTTCCGCTTGG-3′) were used to normalize against plant DNA [23]. Both primer sets were tested for efficiency prior to the qPCR. The qPCR was performed on four plant DNA samples per treatment, by following the manufacturer’s protocol (Power SYBR Green, Life Technologies). To determine bacterial versus plant DNA, we used the formula:

$$ Y={2}^{\left( Ct(rpoB)- Ct(tub)\right)} $$

which is an approximation of the number of bacterial genome copies versus plant genome copy number.

### Strain isolation and culturing

Sonicated and cleaned Arabidopsis root tissue was ground with mortar and pestle in 1 ml phosphate buffer (per litre: 6.33 g of NaH_2_PO_4_·H_2_O, 10.96 g of Na_2_HPO_4_·2H_2_O and 200 μL Silwet L-77). 100 μL of the resulting solution was plated in duplicate on 1/10 TSA and YEM medium plates. 500 μL of the remaining solution was stored in 40% glycerol for later use. In addition, a 100× and 1000× fold dilution were also plated in duplicate. Colonies appearing after 3 days and 7 days were picked (Additional file 2), and stored in YEM medium in 96 well format.

### Genomic DNA isolation for sequencing of the bacterial strains

Strains were inoculated in 10 ml liquid YEM medium. After 5 days of growth, cells were centrifuged at 4000 g, and the medium was discarded. For isolation of DNA, the Qiagen Blood & Tissue kit was used, according to the manufacturer’s instructions. DNA quantity and purity was checked by nanodrop and gel electrophoresis. MOS003 underwent one more round of DNA isolation because the total quantity was not enough to meet the minimal requirements for sequencing.

### Sequencing of the bacterial strains

Sequencing was performed at BGI, using the Illumina HiSeq 2500 PE 150 platform, with a 350 bp insert library size. Paired-end Illumina reads were quality assessed with FastQC [32], and trimmed accordingly with Trimmomatic v. 0.35 [33], and all samples were filtered for a PHRED33 threshold score above 20. Raw reads were assembled with SPAdes v. 3.9.0 [34] under different k-mer sizes with the best assembly judged as containing the smallest number of contigs. Contigs < 1000 bp (only present in MOS002) were removed from the genomes. Assembly statistics were evaluated with QUAST v. 4.5 [35]. The GC content of the contigs revealed contamination in MOS001 and MOS004. The contamination was removed with MaxBin v. 2.2.4 [36]. The ORFs and proteome of the Mossel assemblies were predicted with Prodigal v. 2.6.1 [37]. Assemblies were annotated with the Prokka v1.11 tool [38]. A local BLAST database was made from RefSeq proteins belonging to the *Bradyrhizobium* genus with the BLAST+ package [39]. By specifying the genus flag, Prokka first annotated genes with respect to this database.

### Symbiotic island homology

All publicly available draft and complete assemblies belonging to the *Bradyrhizobium* genus were pulled from NCBI in January 2017. Symbiotic genes were identified with a BLAST reciprocal best hit algorithm [39]. The genes on the symbiotic island of the *B. diazoefficiens* USDA110 assembly was used as the reference for orthologue searching. Presence or absence was defined as a putative orthologue with a minimum 70% identity and 50% coverage as compared to the reference gene.

### Phylogenetic diversity analysis

The 31 AMPHORA genes were found to be present in all the assemblies. The nucleotide sequence of inidividal AMPHORA genes were aligned with Clustal Omega v. 1.2.4 [40] and non-conserved segments were removed with Gblocks [41]. Using the neighbour-joining method in Geneious 8.1, the phylogenetic tree was generated with 1000 bootstraps. The tree was visualized by using Interactive Tree of Life (iTol) platform [42].

### Analyses of functional diversity

The presence or absence of KEGG Orthologue (KO) groups was determined in the ORFs by employing HMM models publically published by Bai et al. [21]. The KO groups were identified using the HMMER v. 3.1b2 [43] hmmsearch tool with thresholds set at an E-value smaller than 10 × 10^− 5^ and larger than 70% coverage. For multiple hits meeting this criteria, only the hit with the smallest E-value was retained. The data was transformed from absolute counts per KO group to either absent or present. Using the *vegdist* command from the Vegan package in R, a distance matrix was created using the binary distance measure. PcoA was performed by the *cmdscale* command of the Stats package in R, with Bray-Curtis as method for dissimilarity.

## Additional files

Additional file 1: **Table S1.** DNA fingerprinting of 102 isolates (XLSX 12 kb)

Additional file 2: **Table S2.** Assembly statistics (XLSX 11 kb)

Additional file 3: **Figure S1.** *Bradyrhizobium* spp. do not affect Arabidopsis shoot fresh weight. Plants were grown for 14 days on sterilised river sand, supplemented with the *Bradyrhizobium* spp. strains, or mock treated (Control). Only shoot weight was measured, roots were used as template for the qPCR in Fig. 5. Each dot represents one replicate (*n* = 30 for each treatment, except for RPG001, for which is *n* = 29). (PDF 6 kb)
