# Supplementary figures and images for: A genetically and functionally diverse group of non-diazotrophic Bradyrhizobium spp. colonizes the root endophytic compartment of Arabidopsis thaliana
Source: BMC Plant Biol. 2018 Apr 11;18:61. doi: 10.1186/s12870-018-1272-y (PMC5896095; doi:10.1186/s12870-018-1272-y)

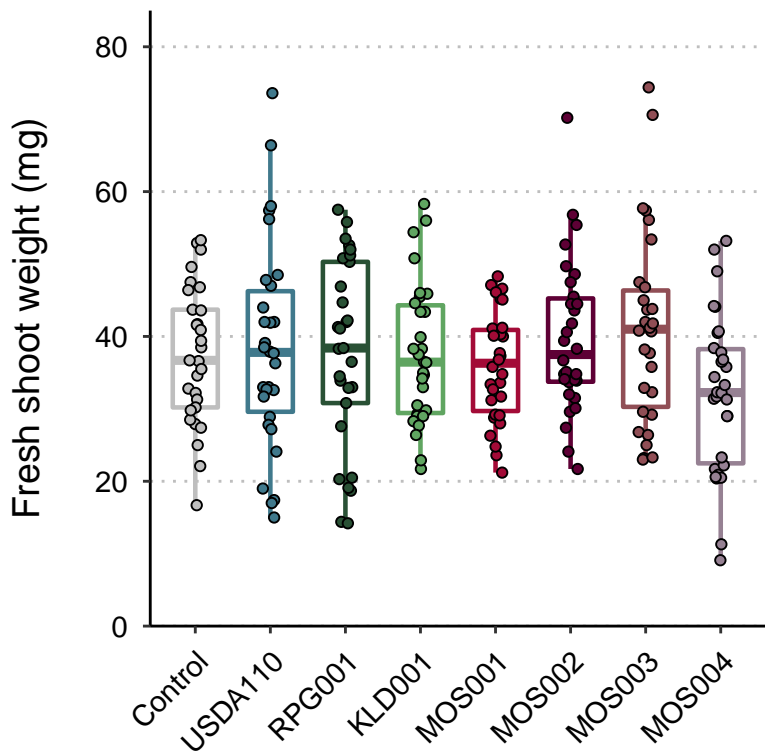

Supplement: Supplementary file 3 — Figure S1. Bradyrhizobium spp. do not affect Arabidopsis shoot fresh weight. Plants were grown for 14 days on sterilised river sand, supplemented with the Bradyrhizobium spp. strains, or mock treated (Control). Only shoot weight was measured, roots were used as template for the qPCR in Fig. 5. Each dot represents one replicate (n = 30 for each treatment, except for RPG001, for which is n = 29). (PDF 6 kb) [file 12870_2018_1272_MOESM3_ESM.pdf]
